# The Association Between Urbanization and Electrocardiogram Abnormalities in China: a Nationwide Longitudinal Study

**DOI:** 10.1007/s11524-023-00816-w

**Published:** 2024-01-12

**Authors:** Jiarun Mi, Xueyan Han, Man Cao, Zhaoyang Pan, Jian Guo, Dengmin Huang, Wei Sun, Yuanli Liu, Tao Xue, Tianjia Guan

**Affiliations:** 1 School of Health Policy and Management, Chinese Academy of Medical Sciences & Peking Union Medical College, Beijing, 100730 China; 2 Department of Cardiology, State Key Laboratory of Complex Severe and Rare Diseases, Peking Union Medical College Hospital, Chinese Academy of Medical Sciences & Peking Union Medical College, Beijing, 100730 China; 3 Medical Research Center, State Key Laboratory of Complex Severe and Rare Diseases, Peking Union Medical College Hospital, Chinese Academy of Medical Sciences & Peking Union Medical College, Beijing, 100730 China; 4 Institute of Reproductive and Child Health, National Health Commission Key Laboratory of Reproductive Health/Department of Epidemiology and Biostatistics, Ministry of Education Key Laboratory of Epidemiology of Major Diseases (PKU), School of Public Health, Peking University Health Science Centre, Beijing, 100191 China; 5 State Environmental Protection Key Laboratory of Atmospheric Exposure, and Health Risk Management and Center for Environment and Health, Peking University, Beijing, 100871 China; 6 Advanced Institute of Information Technology, Peking University, Hangzhou, 311215 China

**Keywords:** Urbanization, Cardiovascular diseases, Electrocardiogram, Longitudinal study, Nighttime light, Impervious surfaces rate

## Abstract

The health effects of urbanization are controversial. The association between urbanization and reversible subclinical risks of cardiovascular diseases (e.g., electrocardiogram (ECG) abnormalities) has rarely been studied. This study aimed to assess the association between urbanization and ECG abnormalities in China based on the China National Stroke Screening Survey (CNSSS). We used changes in the satellite-measured impervious surfaces rate and nighttime light data to assess the level of urbanization. Every interquartile increment in the impervious surfaces rate or nighttime light was related to a decreased risk of ECG abnormalities, with odds ratios of 0.894 (95% CI, 0.869–0.920) or 0.809 (95% CI, 0.772–0.847), respectively. And we observed a U-shaped nonlinear exposure–response relationship curve between the impervious surfaces rate and ECG abnormalities. In conclusion, the current average level of urbanization among the studied Chinese adults remains a beneficial factor for reducing cardiovascular risks.

**Supplementary Information:**

The online version contains supplementary material available at 10.1007/s11524-023-00816-w.

## Introduction

Urbanization reflects the dynamic process of rural-to-urban transition, which is considered to be an important driver of change in human health status [1, 2]. The effect of urbanization on health has been controversial. Some researchers have argued that urbanization brings a number of environmental problems [3] (e.g., air pollution [4] and water pollution [5]), changes the habits of humans [6], and causes mood disorders [7], all of which can impair human health. In contrast, some have argued that urbanization increases accessibility to health services and thus promotes human health [8–10].

Some epidemiological studies have utilized a cross-sectional design to associate different levels of urban development with cardiovascular risk prevalence [11] and mortality [12], most of which suggest that urbanization increases the risk of cardiovascular diseases. However, few studies have analyzed the health effects of urbanization in a longitudinal manner. Additionally, few studies have focused on the subclinical indicators of chronic diseases, such as the estimated glomerular filtration rate (eGFR) [13]. Such studies were helpful to explain the physiological mechanisms underlying the health effects of urbanization and were useful for designing prevention strategies by using subclinical indicators as predictors of relevant diseases. However, specifically for cardiovascular diseases, there is no study on the association between urbanization and predictive subclinical indicators.

Electrocardiogram (ECG) is a clinically common rapid cardiac examination to sensitively reflect the excitation process of the heart, which is of high value for the early detection of arrhythmias [14], ischemic heart disease [15], and coronary heart disease [16]. As a much earlier subclinical cardiovascular outcome compared with other endpoints of cardiovascular diseases (CVDs), such as morbidity and mortality, that were used in previous studies, clarifying the association between urbanization and ECG abnormalities can help us better prevent adverse cardiovascular outcomes and provide an early clinical basis to explore the mechanisms of cardiovascular diseases. In addition, since urbanization is an ongoing dynamic process, adopting a longitudinal design can strengthen the causal correlation between urbanization and the outcome.

Therefore, we applied a longitudinal epidemiological design using the China National Stroke Screening Survey (CNSSS) data to study the long-term association between urbanization and ECG abnormalities in China. The impervious surface rate and nighttime light (NTL) data were used as exposure indicators to measure different urbanization levels.

## Methods

### Study Design and Population

We used a national multicenter longitudinal design to study the long-term association between urbanization and ECG abnormalities among middle-aged and older people in China between 2013 and 2019. The study population was obtained from the China National Stroke Screening Survey (CNSSS), which is an ongoing community-based stroke surveillance program that started in 2013. In brief, under a two-stage stratified sampling framework, the CNSSS used a cross-sectional design that included residents aged no less than 40 years in 31 provinces in mainland China. The details about the methods, design and implementation of CNSSS have been elaborated elsewhere [17, 18]. For this longitudinal study, we included participants with at least two ECG records in the CNSSS by totally excluding 244,805 participants who had only one ECG records in CNSSS.

### Exposure Assessment

Nighttime light (NTL) data were extracted from a global-scale NTL time-series dataset (1992–2018) that was formed by integrating the intercalibrated NTL observations from the Defense Meteorological Satellite Program (DMSP) data (1992–2013) and the simulated DMSP-like NTL observations from the Visible Infrared Imaging Radiometer Suite (VIIRS) data (2012–2018). The details of this dataset are described elsewhere [19].

Impervious surfaces are the major part of human settlements, including rooftops, compacted soils, and pavements, which are mainly composed of artificial components impeding the natural infiltration process of water into soil [20, 21]. We obtained the impervious surfaces from Global Land Cover, which was generated by previously verified and reliable impervious surfaces mapping algorithms and the Google Earth Engine (GEE) platform. For data with a resolution of 30 m, Gong et al. (2019) evaluated the accuracy of the data and found that the overall accuracy of the data was greater than 90% [22].

CNSSS included the community-level address records (latitude and longitude) of participants. Owing to the restricted availability of detailed residential data, we collected merely the community-level addresses for each participant both at the initial stage and during the subsequent follow-up. Subsequently, we mapped all individuals within the administrative boundaries of their respective communities as delineated in a 2010 geographical map. The two exposure indicators were aggregated within a regular 10 km × 10 km grid to be matched with the addresses in CNSSS because the 1 km × 1 km or 30 m × 30 m scale of grids employed in our study might not comprehensively encompass all community areas and their immediate surroundings. The original spatial resolution of NTL data was 1 km × 1 km, and we averaged the values within each 10 km × 10 km grid. Impervious surface was a binary indicator (0 or 1) within a 30-m × 30-m scale. Similarly, we aggregated the binary values within each 10 km × 10 km grid to derive a percentage of each grid (i.e., impervious surfaces rate).The Kendall’s tau coefficient between impervious surfaces rate and nighttime light was calculated to be 0.143, substantiating the weak correlation between impervious surfaces rate and NTL. The two urbanization indicators allowed for vary over time, and their distributions were shown in Fig. 2 with box plots.

### Outcome and Covariates

An observed abnormal electrocardiogram diagnosed by a well-trained physician of level II or above hospitals with a 12-lead electrocardiogram was the outcome of this study. ECG abnormalities, which often signal cardiac conditions, can present as QRS wave abnormalities, *P* wave abnormalities, ST segment abnormality, arrhythmia, etc. In particular, ECG abnormalities among older adults were associated with an significantly increased risk of coronary heart disease [16]. We collected personal information using a face-to-face structured questionnaire. This questionnaire included (1) sociodemographic variables: sex, age, height, and weight; (2) lifestyle variables: smoking, alcohol consumption, milk intake, and physical activity; and (3) medical history variables: previous diagnosis of atrial fibrillation (AF), dyslipidemia, hypertension, and diabetes mellitus. We also calculated body mass index (BMI) using the above variables.

### Statistical Analysis

In descriptive analysis, we used chi-square tests and *t* test to compare the covariate distributions of participants with normal versus abnormal ECG records at baseline. We used Mann‒Whitney tests to compare the differences in the exposure levels.

A generalized estimating equation (GEE) model was used to measure the association between urbanization and ECG abnormalities, which is a semiparametric and quasilikelihood estimation method based on a generalized linear model, and it has been widely adopted in longitudinal modeling [23–25]. In addition, we used multiple imputation to fill in the missing values of covariates (sex, age, height, weight, smoking, alcohol consumption, milk intake, physical activity, previous diagnosis of atrial fibrillation (AF), dyslipidemia, hypertension, and diabetes mellitus) with the help of the R package *mice*. For continuous variables, we employed predictive mean matching (PMM) as the imputation method. For binary variables, logistic regression was utilized, and for both ordinal and nominal categorical variables, we adopted polynomial regression. The imputation process involved five iterations to ensure the robustness and accuracy of the imputed data. Given that participants with baseline ECG abnormalities tended to be missed due to a higher risk of death, participants who attended at least one follow-up survey could have a better ECG performance at baseline than those who had only one ECG record. Therefore, the inverse probability weight (IPW) approach was adopted to avoid this bias. The IPW score was calculated by a logit model, which regressed the involvement status of this study (i.e., inclusion/exclusion) against the baseline ECG performance and covariates of all participants. The sample size of IPW was 39,449 participants, including 82,926 screening records. We controlled for age, seasonality (namely, Dec–Feb, Mar–May, Jun–Aug, and Sep–Nov), lifestyle factors (namely, smoking, alcohol drinking, milk intake, and physical inactivity), medical history factors (namely, AF, dyslipidemia, hypertension, and diabetes), and BMI in the main model (i.e., Model 5) and used the exposure time window of 2 years in the main analysis. Considering the relevance between socioeconomic conditions and urbanization, we also controlled for the annual gross regional product per capita at the provincial level in model 5.

Moreover, we removed IPW from our models and repeated the above analysis using different exposure time windows from 0 to 0–2 years to evaluate the robustness of the association. We conducted subgroup analyses to explore the different associations between urbanization and ECG abnormalities among region, age group, sex, AF at baseline, dyslipidemia, hypertension, diabetes mellitus, smoking, drinking, physical inactivity, drinking milk, and BMI. The heterogeneity between subgroups was assessed using the Wald test. In light of the complicated association between urbanization and ECG abnormalities, we used a set of penalized spline functions to model the nonlinear exposure–response curve with the help of R package *mgcv*. All statistical analyses were performed using the R software (version 4.2.0). In the main analyses, statistical tests were two-sided, and a *p* value of < 0.05 was considered to indicate statistical significance. In the subgroup analyses, a *p* value of < 0.01 was considered to be statistically significant.

## Results

### Descriptive Analyses

Table 1 shows the baseline characteristics of the 39,449 participants included in our study. The mean age of all participants was 60.1 years with a standard deviation of 10.3 years. Among all participants, 2.8% had atrial fibrillation at baseline, 26.7% had dyslipidemia, 46.5% had hypertension, and 14.3% had diabetes mellitus. Women accounted for 54.2%, and 24.0% of participants had ECG abnormalities at the baseline survey. The median impervious surface rate was 23.58% (interquartile range: 8.15–43.29%), and the median NTL was 36.70 (interquartile range: 12.35–56.56) digital numbers in all participants.

**Table 1:** Baseline sample characteristics of 39,449 participants included in our study

Variables	ECG normalities (*N* = 29,989)	ECG abnormalities (*N* = 9460)	Overall (*N* = 39,449)	*P*-value
Impervious surfaces rate, median (IQR)	25.98 (9.38, 46.82)	16.61 (7.00, 37.80)	23.58 (8.15, 43.29)	** < 0.001**
Nighttime light, median (IQR)	38.73 (13.42, 57.29)	32.43 (10.67, 52.60)	36.70 (12.35, 56.56)	** < 0.001**
Age group				** < 0.001**
[40,50)	6401 (21.3%)	927 (9.8%)	7328 (18.6%)	
[50,60)	8635 (28.8%)	2282 (24.1%)	10,917 (27.7%)	
[60,70)	8868 (29.6%)	3525 (37.3%)	12,393 (31.4%)	
≥ 70	4268 (14.2%)	2374 (25.1%)	6642 (16.8%)	
Missing	1817 (6.1%)	352 (3.7%)	2169 (5.5%)	
Sex				**0.504**
Male	12,067 (40.2%)	3837 (40.6%)	15,904 (40.3%)	
Female	16,110 (53.7%)	5271 (55.7%)	21,381 (54.2%)	
Missing	1812 (6.0%)	352 (3.7%)	2164 (5.5%)	
Atrial fibrillation at baseline				** < 0.001**
No	29,989 (100%)	8342 (88.2%)	38,331 (97.2%)	
Yes	0 (0%)	1118 (11.8%)	1118 (2.8%)	
Dyslipidemia				** < 0.001**
No	18,221 (60.8%)	4247 (44.9%)	22,468 (57.0%)	
Yes	7383 (24.6%)	3158 (33.4%)	10,541 (26.7%)	
Missing	4385 (14.6%)	2055 (21.7%)	6440 (16.3%)	
Hypertension				** < 0.001**
No	17,430 (58.1%)	3671 (38.8%)	21,101 (53.5%)	
Yes	12,549 (41.8%)	5787 (61.2%)	18,336 (46.5%)	
Missing	10 (0.0%)	2 (0.0%)	12 (0.0%)	
Diabetes mellitus				** < 0.001**
No	22,618 (75.4%)	6825 (72.1%)	29,443 (74.6%)	
Yes	4021 (13.4%)	1614 (17.1%)	5635 (14.3%)	
Missing	3350 (11.2%)	1021 (10.8%)	4371 (11.1%)	
Smoke				** < 0.001**
No	19,359 (64.6%)	5799 (61.3%)	25,158 (63.8%)	
Yes	6015 (20.1%)	2279 (24.1%)	8294 (21.0%)	
Missing	4615 (15.4%)	1382 (14.6%)	5997 (15.2%)	
Drink				** < 0.001**
No	25,389 (84.7%)	7759 (82.0%)	33,148 (84.0%)	
Yes	4384 (14.6%)	1643 (17.4%)	6027 (15.3%)	
Missing	216 (0.7%)	58 (0.6%)	274 (0.7%)	
Physical inactivity				** < 0.001**
No	10,199 (34.0%)	4082 (43.2%)	14,281 (36.2%)	
Yes	19,622 (65.4%)	5337 (56.4%)	24,959 (63.3%)	
Missing	168 (0.6%)	41 (0.4%)	209 (0.5%)	
Milk				**0.358**
No	17,863 (59.6%)	5264 (55.6%)	23,127 (58.6%)	
Yes	3778 (12.6%)	1054 (11.1%)	4832 (12.2%)	
Missing	8348 (27.8%)	3142 (33.2%)	11,490 (29.1%)	
BMI^a^				** < 0.001**
Underweight	455 (1.5%)	188 (2.0%)	643 (1.6%)	
Normal	11,769 (39.2%)	3289 (34.8%)	15,058 (38.2%)	
Overweight	12,541 (41.8%)	3970 (42.0%)	16,511 (41.9%)	
Obese	5177 (17.3%)	1959 (20.7%)	7136 (18.1%)	
Missing	47 (0.2%)	54 (0.6%)	101 (0.3%)	

^a^BMI: body mass index

### Association Between Urbanization and ECG Abnormalities

**Fig. 1:**
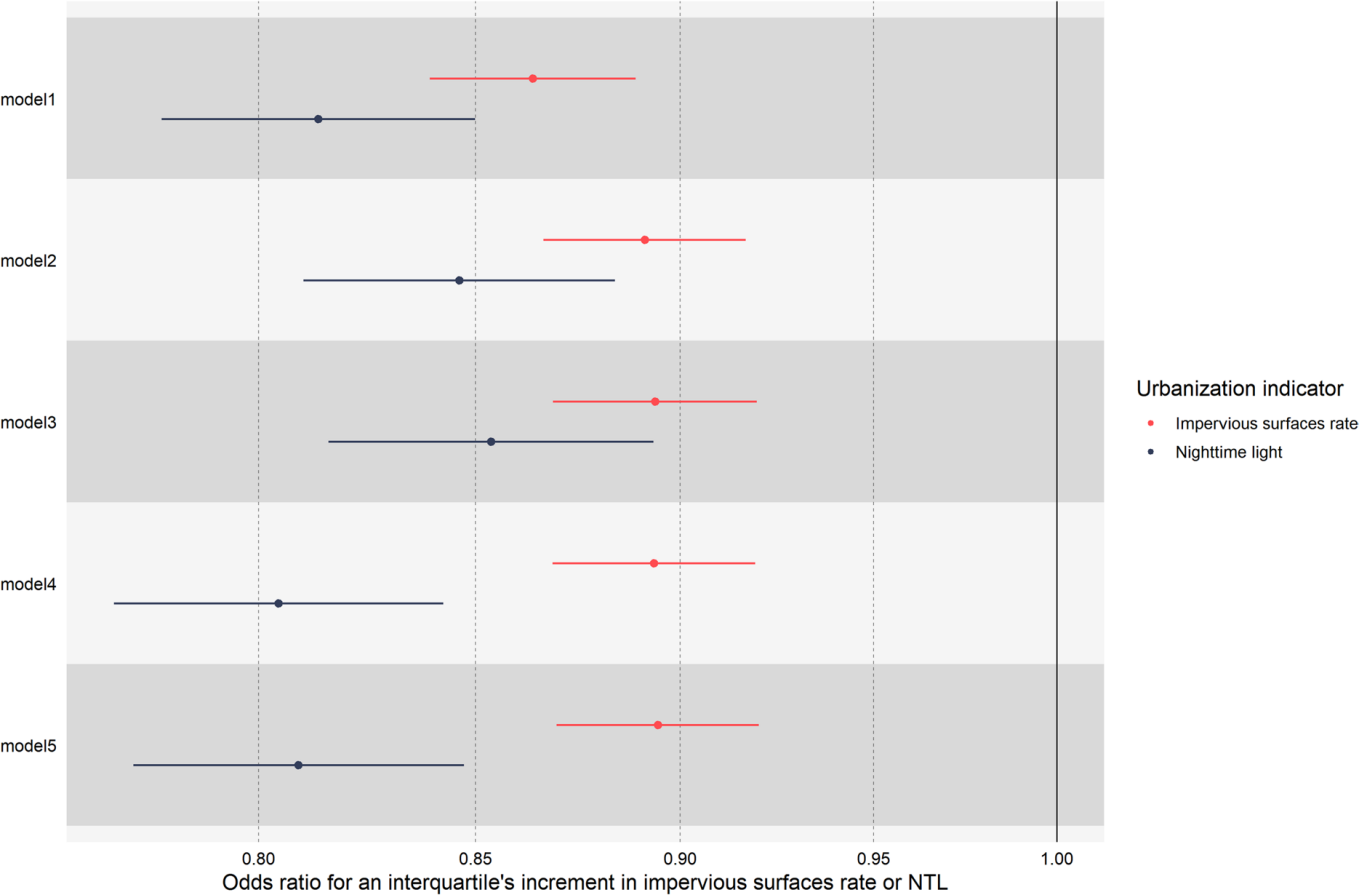
The association between urbanization and ECG abnormalities estimated by different models. Model 1 was only adjusted for age by using the natural spline function. Model 2 was additionally adjusted for seasonality. Model 3 was additionally adjusted for lifestyle factors (namely, smoking, alcohol consumption, milk consumption, physical inactivity, and BMI). Model 4 was additionally adjusted for medical history factors (namely, AF, dyslipidemia, hypertension, and diabetes mellitus). Model 5 was additionally adjusted for the annual Gross Regional Product per Capita at the provincial level

Urbanization was associated with a decreased risk of ECG abnormalities. As is shown in Fig. 1, based on the full adjusted linear model (model 5), the odds ratio of ECG abnormalities was estimated as 0.894 (95% CI, 0.869–0.920) and 0.809 (95% CI, 0.772–0.847) for per interquartile increment in impervious surfaces rate and NTL, respectively. The interaction between urbanization and time was found to be significant. Specifically, for each 1-year increase in time, and with an interquartile increment in impervious surfaces rate and NTL, the odds of the protective effect were 1.063 and 1.061 times higher (95% CI, 1.042–1.085 or 1.029–1.094), respectively. This suggests that the impact of urbanization on ECG abnormalities becomes more pronounced over time. We found no change in the significance of the model estimates by removing IPW and using different exposure time windows (Fig. S1-S2). The results confirmed the robustness of the estimated associations.

### Nonlinear Exposure–Response Association

**Fig. 2:**
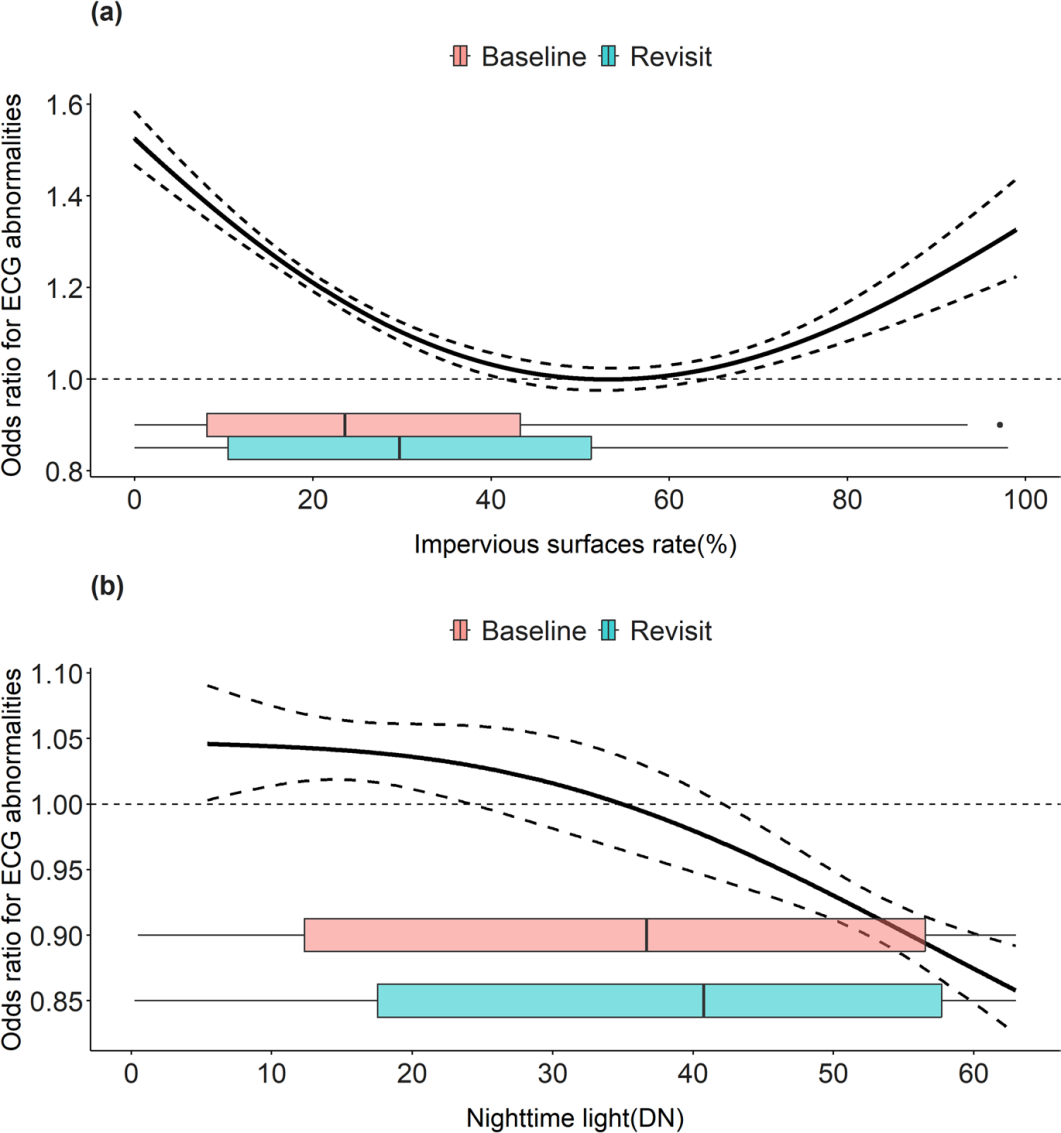
The nonlinear exposure–response relationship between urbanization and ECG abnormalities. The dashed line represents the 95% confidence intervals, and the distributions of the impervious surfaces rate and nighttime light data of the baseline and revisit populations are shown in the box plot

The nonlinear exposure–response curves between the two urbanization indicators and ECG abnormalities are shown in Fig. 2. A U-shaped curve was found for the impervious surfaces rate, while a monotonically decreasing curve was found for NTL. The exposure level for the minimum risk of ECG abnormalities was approximately an impervious-area rate of 55%, according to the U-shaped exposure–response function. The odds ratio of ECG abnormalities was estimated as 1.521 (95% CI, 1.465, 1.580) and 1.119 (95% CI, 1.079, 1.160) in areas with 5% quantile (impervious surfaces rate, 0.22%) and 95% quantile (impervious surfaces rate, 79.31%) of baseline impervious surface rate when using 55% as a reference impervious-area rate. Compared to impervious areas, NTL was distributed in a left-skewed pattern, which made NTL less sensitive to high-level urbanization. Therefore, the results based on NTL data might only show the health effect of insufficient urbanization.

### Subgroup Analysis

Fig. S3-S4 show the results of subgroup analyses on the association between urbanization and ECG abnormalities. In participants with hypertension and a baseline AF, the protective effect of urbanization on ECG abnormalities was reduced, even to the extent of making urbanization a risk factor for ECG abnormalities among a certain subpopulation. For instance, among the participants with an AF at baseline, the odds ratio of ECG abnormalities was 1.243 (95% CI, 0.978–1.581) for an interquartile increment in NTL, although the effect was not statistically significant. In contrast, among healthy participants, the odds ratio was estimated as 0.803 (95% CI, 0.766–0.841). Additionally, we found that urbanization in Northeast and South China was associated with an increased risk of ECG abnormalities, which was different from the estimates in other regions. For every interquartile increment in impervious surfaces rate, the odds ratio of ECG abnormalities was 1.071 (95% CI, 1.024–1.120) and 1.152 (95% CI, 1.017–1.306) in Northeast and South China, respectively. The estimates based on impervious area showed similar patterns for the spatially varied effect of urbanization on ECG abnormalities.

## Discussion

This study is the first to use national longitudinal data to assess the association between urbanization and ECG abnormalities. Based on the GEE estimates of the impervious surfaces rate and NTL, we confirmed that urbanization was significantly associated with a reduced risk of ECG abnormalities. However, this association was reduced or reversed significantly among participants with hypertension and AF, and the association between ECG and urbanization varied significantly among participants in different regions. In addition, we found a U-shape in the nonlinear exposure–response curve of the impervious surfaces rate, indicating the adverse effect of urbanization levels that are too low and too high on ECGs.

ECG abnormalities are a predictive indicator of CVDs, which are the leading cause of death worldwide [26]. The burden of CVDs in China is also severe. The morbidity, prevalence, and mortality of CVDs among the population in China have been on a rapid rise in the last 30 years when ischemic heart disease, as well as stroke, became the leading causes of death and disability-adjusted life years [27]. Meanwhile, unlike developed countries that have entered a steady phase of high-level urbanization, China is still experiencing a rapid process of urbanization. The robust association between urbanization and ECG abnormalities obtained in this study could be helpful for the surveillance and prevention of CVDs in the context of rapid urbanization. Overall, we found that urbanization remains a beneficial factor for better ECG performance. Because the CVD mortality in rural areas has exceeded that in urban areas since 2009 in China [28], our result was consistent with expectations.

While the relationship between urbanization and ECG abnormalities has not been studied previously, some epidemiological studies using other subclinical indicators or biomarkers of CVDs can support our findings. For instance, compared with others living in urban areas, a cross-sectional study in China found a significantly higher risk of CVDs among children living in rural areas as well as increased cardiorespiratory fitness levels (OR: 2.04, 95% CI: 1.29–3.25), metabolic risk scores (OR: 2.33, 95% CI: 0.50–3.62), and triglyceride values (OR: 2.40, 95% CI: 1.62–3.57) [29]. Additionally, a lower level of cardiovascular risk factors and surrogate markers of CVDs, such as left ventricular mass and intima-media thickness, was found among urban participants in a study conducted in Finland [30].

Some longitudinal studies showed a similar nonlinear exposure–response relationship. A U-shaped nonlinear exposure–response relationship curve between urbanization and poststroke disability was displayed in one study [31]. Since stroke is a serious complication of some specific types of ECG abnormalities [32], this finding could support our U-shaped impervious surfaces rate curve. In addition, a study of cardiovascular biological risk trajectories of middle-aged and elderly Chinese participants found that urban‒rural differences in cardiovascular risk changed gradually from middle to later life and that urban residents exhibited higher cardiovascular risk in mid-to-late life than rural residents [33]. The U-shaped curve means that both too high and too low levels of impervious surfaces rates are adverse to ECG outcomes. On the one hand, an increase in urbanization implies economic growth and better availability of medical resources. However, people living in areas with higher levels of urbanization are exposed to more risk factors for CVDs, including air pollution, noise, and mental stress [34]. The threshold suggested by the U-shaped curve may be helpful to estimate the optimal urbanization level for reasonable monitoring and control of the urbanization process to minimize the risk of ECG abnormalities.

The regional difference was shown in the subgroup analyses. Our results suggested that the positive association between urbanization and ECG conditions was reversed in Northeast and South China compared with other regions. This regional difference in ECG abnormalities might provide evidence for optimizing prevention against CVDs by targeting the areas with the highest need.

Given that AF is the most common ECG-diagnosed cardiac arrhythmia [35], the negative association between urbanization and ECG conditions among participants with AF history was credible. According to a recent national study, China is bearing a huge AF burden, and the AF prevalence is increasing rapidly [36]. Therefore, the identification of the negative association among the AF subgroup had some clinical and public health implications in China. In addition, a strong correlation between hypertension and specific types of ECG abnormalities or cardiovascular outcomes has been found in previous studies [36, 37]. The reduced protective effect shown in the hypertension subgroup in this study could support such previous findings.

Considering the differences in the level of urbanization within different cities or different rural areas, it is inaccurate to simply define the urban‒rural difference in health outcomes as an urbanization health effect. Therefore, unlike most previous studies using a binary variable (urban/rural) as an equivalent of urbanization, we used two continuous indicators from satellite remote sensing to measure different urbanization levels more comprehensively. Currently, the impervious surface rate is recognized as the most direct representation of the urbanization level and one of the main indicators of human settlements [38]. Previous studies have shown that NTL is closely related to human economic and social activities and can even be used to estimate the gross domestic product (GDP) [39]. As the two indicators measured urbanization from different aspects, differences in individual results are acceptable in this study. Poverty in urban areas might explain the adverse health effect of urbanization shown in the nonlinear curve, but it was not reflected by satellite NTL data.

Our study has the following limitations. First, although we used a longitudinal epidemiological design, the participants in each survey were obtained from a repeat sample of CNSSS, and no follow-up survey was conducted for the entire population of CNSSS participants. We excluded individuals without follow-up records, which may have produced selection bias, and we subsequently applied IPW to try to overcome that bias. Second, we did not consider other environmental exposures associated with rapid urbanization, such as temperature, air pollution, and noise. These exposures may be important mediators between urbanization and ECG abnormalities. Future studies could be supplemented with environmental exposure associated with urbanization to study the mechanisms of urbanization effects on ECG abnormalities. Third, although a negative impact of urbanization was found in the AF subgroup, we did not add AF as a secondary health outcome in the main analysis. The correlation between specific types of ECG abnormalities (e.g., AF, ischemic change and left ventricular hypertrophy) and urbanization should be focused on in the future.

## Conclusion

This study reveals a complex association between urbanization and ECG abnormalities. The current average level of urbanization in China remains a beneficial factor in reducing cardiovascular risks. Based on the U-shaped relationship, the optimal level of urbanization can be estimated to guide the prevention of cardiovascular diseases.

### Supplementary Information

Below is the link to the electronic supplementary material.

Supplementary file1 (DOCX 649 KB)

## Data Availability

The authors do not have permission to share data.

## Declarations

### Submission Declaration and Verification

The manuscript has not been published elsewhere in part or entirely and is not under consideration by another journal. All authors have approved the enclosed manuscript.
