# Supplementary material for: The Association Between Urbanization and Electrocardiogram Abnormalities in China: a Nationwide Longitudinal Study
Source: J Urban Health. 2024 Jan 12;101(1):109–19. doi: 10.1007/s11524-023-00816-w (PMC10897075; doi:10.1007/s11524-023-00816-w)
Supplement: Supplementary file 1 — Supplementary file1 (DOCX 649 KB) [file 11524_2023_816_MOESM1_ESM.docx]

# Supplementary Information

**The association between urbanization and electrocardiogram abnormalities in China: a nationwide longitudinal study**

Jiarun Mi, Xueyan Han, Man Cao, Zhaoyang Pan, Jian Guo, Dengmin Huang, Wei Sun, Yuanli Liu, Tao Xue^*^, Tianjia Guan^*^

*** Corresponding author:** Tianjia Guan, Tao Xue

Tianjia Guan* — School of Health Policy and Management, Chinese Academy of Medical Sciences & Peking Union Medical College, Beijing, China, 100730; Email: [gtj@sph.pumc.edu.cn](mailto:gtj@sph.pumc.edu.cn)

Tao Xue* — Institute of Reproductive and Child Health/ National Health Commission Key Laboratory of Reproductive Health and Department of Epidemiology and Biostatistics, School of Public Health, Peking University, Beijing, China, 100191; Email: [txue@hsc.pku.edu.cn](mailto:txue@hsc.pku.edu.cn)


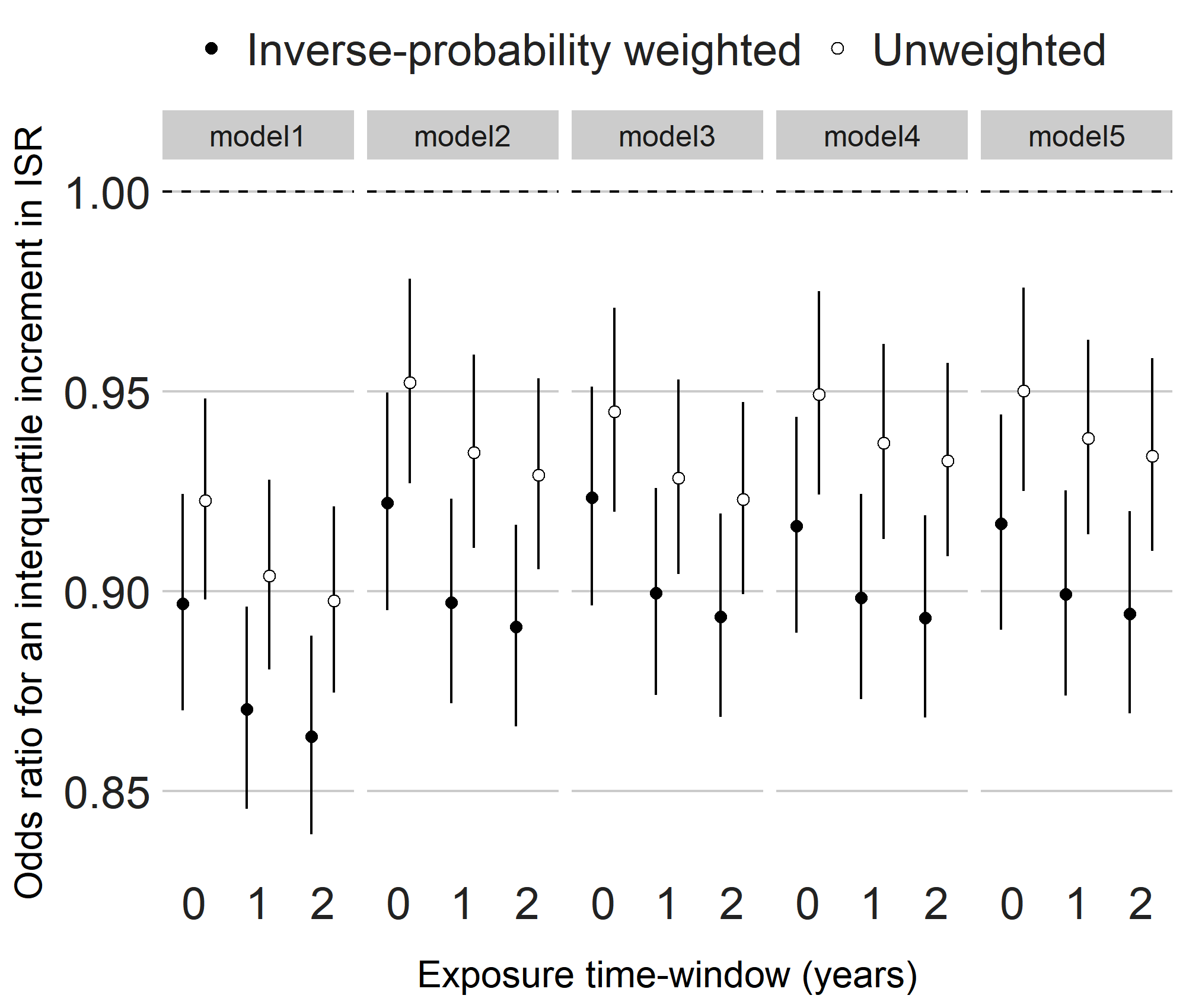


**Fig. S1** The association between an interquartile increment in impervious surfaces rate and ECG abnormalities estimated by using different exposure time-windows


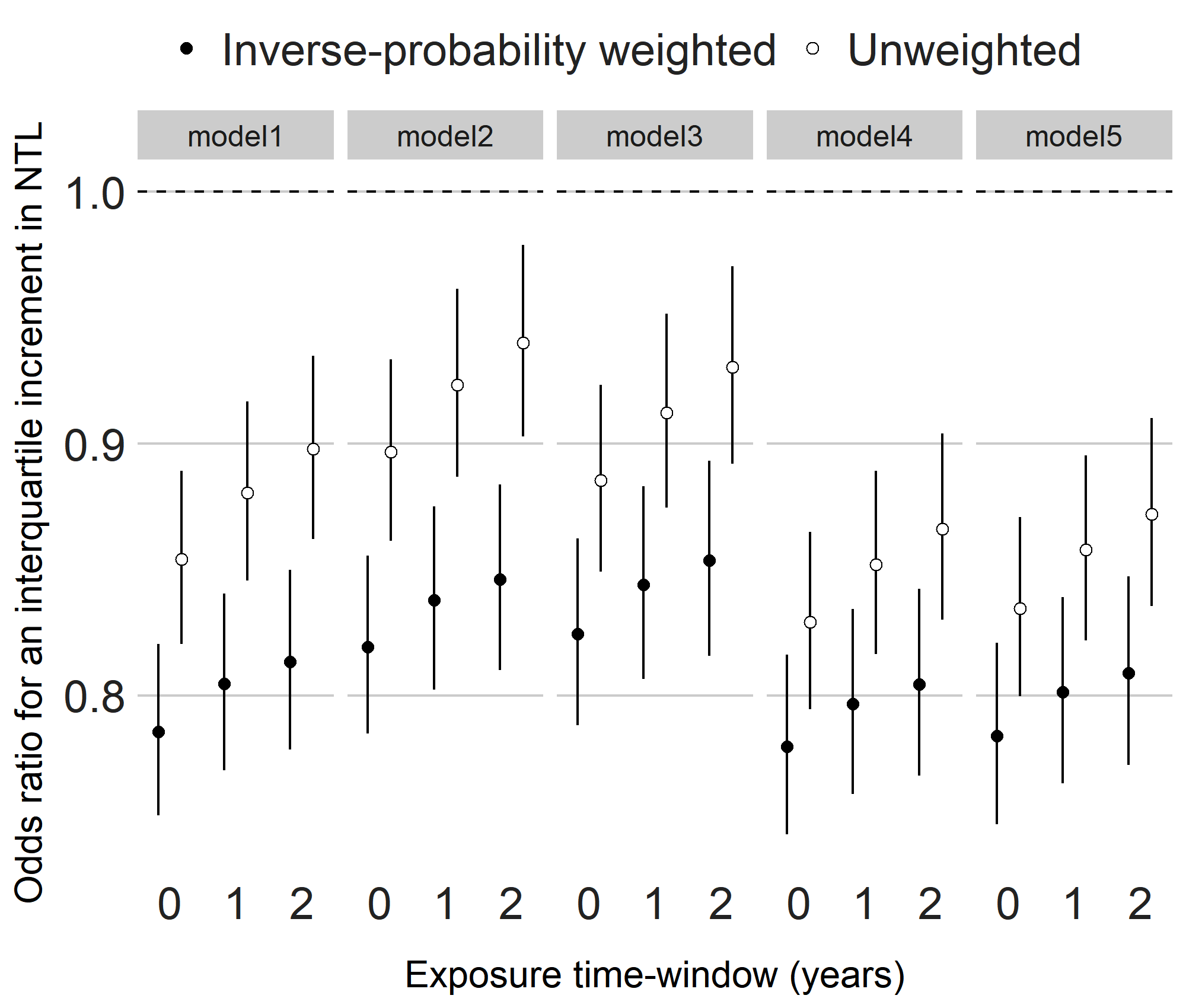


**Fig. S2** The association between an interquartile increment in NTL and ECG abnormalities estimated by using different exposure time-windows


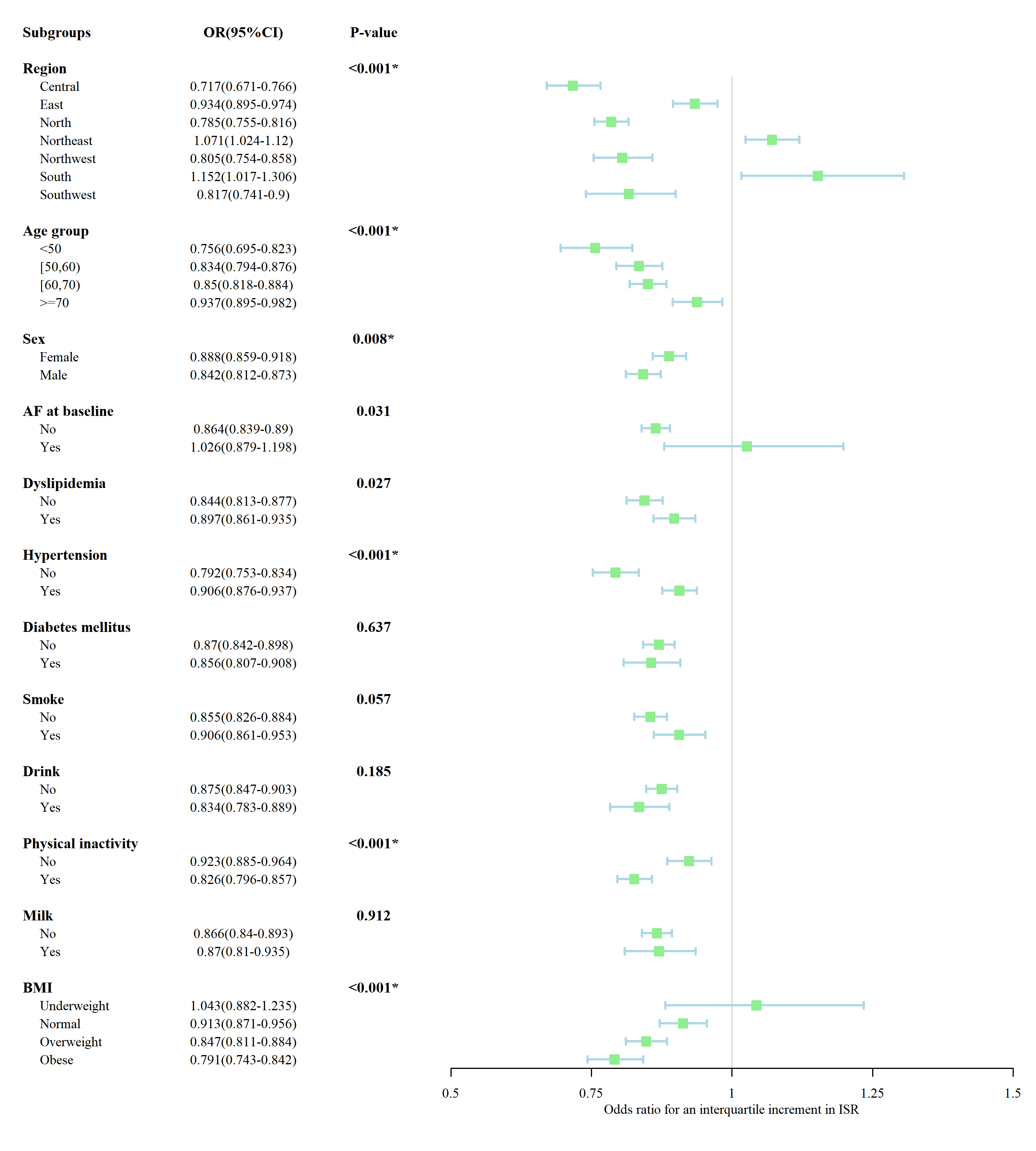


**Fig. S3** Subgroup analyses of the association between an interquartile increment in impervious surfaces rate and ECG abnormalities (P-value < 0.001 was considered as statistically significant in subgroup analyses and was marked with *)


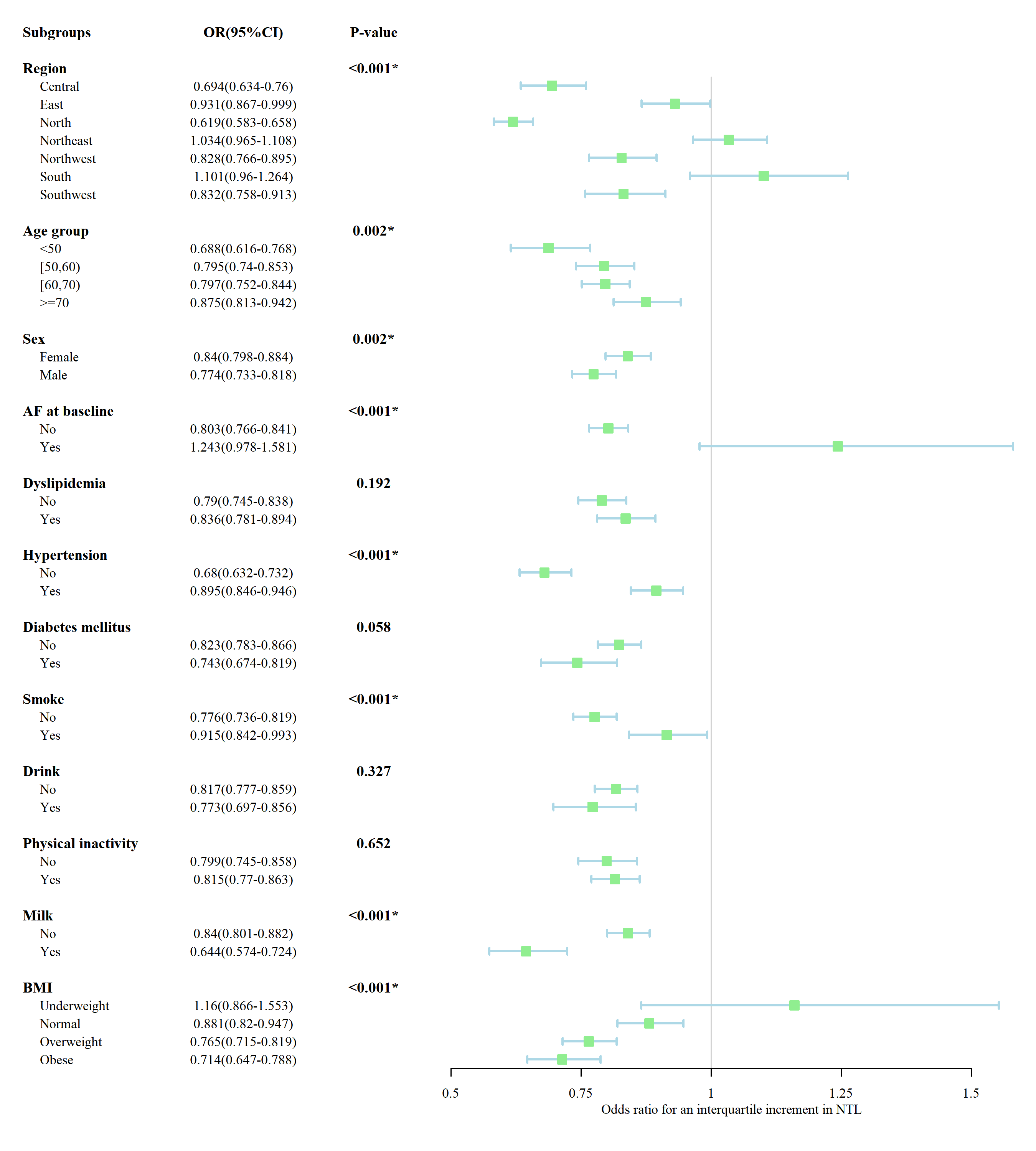


**Fig. S4** Subgroup analyses of the association between an interquartile increment in NTL and ECG abnormalities (P-value < 0.001 was considered as statistically significant in subgroup analyses and was marked with *)
